# Smart Bone Graft Composite for Cancer Therapy Using Magnetic Hyperthermia

**DOI:** 10.3390/ma15093187

**Published:** 2022-04-28

**Authors:** Geovana L. Santana, Murilo C. Crovace, Ernesto E. Mazón, Adilson J. A. de Oliveira, Theo Z. Pavan, Edgar D. Zanotto

**Affiliations:** 1 Vitreous Materials Laboratory (LaMaV), Department of Materials Engineering, Federal University of São Carlos, São Carlos 13565-905, SP, Brazil; mcc@ufscar.br; 2 Graduate Program in Materials Science and Engineering (PPGCEM), São Carlos 13565-905, SP, Brazil; 3 Department of Physics, FFCLRP, University of São Paulo, Ribeirão Preto 14040-901, SP, Brazil; mazon_valadez@hotmail.com (E.E.M.); theozp@gmail.com (T.Z.P.); 4 Department of Physics, Federal University of São Carlos, São Carlos 13565-905, SP, Brazil; adilson@df.ufscar.br

**Keywords:** bone cancer, hyperthermia, smart material, bioactive glass, magnetic composite

## Abstract

Magnetic hyperthermia (MHT) is a therapy that uses the heat generated by a magnetic material for cancer treatment. Magnetite nanoparticles are the most used materials in MHT. However, magnetite has a high Curie temperature (*Tc*~580 °C), and its use may generate local superheating. To overcome this problem, strontium-doped lanthanum manganite could replace magnetite because it shows a *Tc* near the ideal range (42–45 °C). In this study, we developed a smart composite formed by an F18 bioactive glass matrix with different amounts of Lanthanum-Strontium Manganite (LSM) powder (5, 10, 20, and 30 wt.% LSM). The effect of LSM addition was analyzed in terms of sinterability, magnetic properties, heating ability under a magnetic field, and in vitro bioactivity. The saturation magnetization (M_s_) and remanent magnetization (M_r_) increased by the LSM content, the confinement of LSM particles within the bioactive glass matrix also caused an increase in *Tc*. Calorimetry evaluation revealed a temperature increase from 5 °C (composition LSM5) to 15 °C (LSM30). The specific absorption rates were also calculated. Bioactivity measurements demonstrated HCA formation on the surface of all the composites in up to 15 days. The best material reached 40 °C, demonstrating the proof of concept sought in this research. Therefore, these composites have great potential for bone cancer therapy and should be further explored.

## 1. Introduction

Cancer is the second leading cause of death worldwide, after heart disease, and is, thus, an important barrier to increasing life expectancy. This disease typically initiates due to mutations in genes that result in abnormal cell division and growth [1]. There are more than 100 different types, one of which is bone cancer [2]. According to the American Cancer Society, the occurrence of more than 3600 new cases of bone cancer is estimated for 2021 in the US [3]. Osteosarcoma is the most common type of primary bone cancer, followed by chondrosarcoma and Ewing sarcomas. The treatment is usually based on surgical removal of a tumor, followed by or combined with complementary treatments, such as chemotherapy, radiotherapy, hyperthermia and immunotherapy [4]. Most of these therapies provide solutions that are not selective enough because they destroy not only the malignant tumor, but also many healthy cells. Thus, a great challenge is to develop a therapy that cures this potentially fatal disease with minimum side effects [5,6].

Hyperthermia (HT) is a type of cancer therapy, in which the cancer cells are heated by external agents to a temperature of ~43 °C. This induces cell death by destructing proteins and inhibits the formation of new blood vessels, with little or no harm to normal tissue. This therapy varies according to the heat source and can be used as adjunctive treatment associated with radiotherapy and chemotherapy. The approach adopted to increase the local temperature using an external alternating current magnetic field (AMF) is called magnetic hyperthermia (MHT). MHT uses the heat response generated by magnetic particles when subjected to a magnetic field, forming an internal heat source without any chemical substances or severe toxicity [7,8]. 

MHT science has been growing, although several challenges are still being discussed by the scientific community for clinical applications, such as: (a) thermal conversion efficiency; MHT devices should have the capacity of accurately delivering high thermal energy using a low mass of magnetic particles; (b) the field frequency and magnitude should be selected to minimize the production of eddy currents and dielectric heating, which can generate undesirable nervous and muscle responses; (c) the magnetic particles can be administered by different routes, such as intravenous, subcutaneous, intratumoral (surgically or not), or oral administration. For all these cases, these particles should be formed by biologically inert or bioactive support (liquid or solid) necessary to make them compatible within the body; (d) the magnetic material must be precisely controlled to prevent local overheating or heterogeneous temperature distribution in tumor mass and ensure the heat transference to local treatment [8,9,10].

Superparamagnetic and ferromagnetic iron oxide particles (Fe_3_O_4_) are usually considered as candidates for MHT due to their adequate magnetic properties, such as high specific absorption rate (*SAR*), high saturation magnetization, and biocompatibility; however, one drawback of these materials is their far too high Curie temperature, which can reach 585 °C. Magnetic-field-induced heating occurs when the material presents magnetic order. Above *Tc*, the material changes to a non-ordered state, then it no longer responds (thermally) to the external field. High Curie temperatures give rise to uncontrolled and non-uniform tumor heating, which, in turn, may destroy the healthy adjacent tissues [11,12].

To achieve a ‘self-controlled’ temperature and avoid local overheating, materials that present a *Tc* within the MHT temperature range of interest have been studied. In this view, lanthanum-strontium manganites (LSM) are of particular interest. The LSM crystalline phases are manganese oxide-based compounds (manganites), with the formula R_(1−x)_A_x_MnO_3_, where the R sites are substituted by the rare earth metal–lanthanum, and A by strontium. In the case of LSM, the *Tc* can be tailored within the temperature range of interest by cationic (x) composition variations that influence distortions and the mixed valence of manganese. It is also possible to change the characteristic temperature depending on several parameters, such as phase composition, particle size and shape, particle arrangement, and ac-field frequency [13,14]. Recent review papers have been published summarizing the physicochemical and magnetic properties of these materials, influenced by the synthesis methods and reaction conditions, as well as by the microstructural parameters, such as particle size, surface coating (nature/amount), stoichiometry, concentration and/or applied AMFs (including magnetic field (H) and frequency (f)) in MHT application [15,16]. In the case of osteosarcoma, for example, it is common for a tumor to be surgically removed, leaving a significant bone defect behind, which must be filled with a graft. Besides the massive bone lesion, osteosarcoma has a high recurrence rate, requiring additional treatments. Thus, a bone graft having a balance between bioactivity and magnetic properties would be highly desirable. In this work, we studied the effect of La_0.8_Sr_0.2_MnO_3_ (LSM) additions in powder form in the sinterability and in vitro bioactivity of F18 bioactive glass, as well as the magnetic properties of the composites obtained. Our purpose was to develop a smart composite material having a double function: (1) to regenerate the bone tissue after tumor removal and (2) to kill remaining or recurrent cancer cells if needed, having the advantage of not overheating the neighboring healthy cells.

## 2. Materials and Methods

### 2.1. Materials

Lanthanum Oxide III (La_2_O_3_, 99.99%), Strontium Carbonate (SrCO_3_, 99.9%) and Manganese Oxide III (Mn_2_O_3_, 99%) were purchased from Sigma-Aldrich (São Paulo, SP, Brazil).

### 2.2. Preparation of Materials

#### 2.2.1. Preparation of LSM

LSM particles were synthesized by a solid-state reaction technique. According to previous work [17,18,19,20], samples were prepared using mixtures of La_2_O_3_, Mn_2_O_3_, and SrCO_3_ in the cation molar ratio of 0.8La:0.2Sr:1Mn. They were homogenized in a planetary ball mill (Pulverisette 6–FRITSCH) at 350 rpm for 60 min, with anhydrous isopropyl alcohol. The slurry was dried at 100 °C/24 h and the resulting powder was uniaxially pressed into ø = 10 mm discs, using a pressure of ~200 MPa. These discs were pre-sintered in an electric muffle furnace at 1300 °C for 3 h, with a heating rate of 10 °C/min. After cooling, these discs were fragmented by a mortar agate until the particles passed through a 1mm sieve and, following that it was milled with isopropyl alcohol in a planetary mill for 30 min, with a rotating speed of 350 rpm reaching the average particle size of 1.7 μm. 

#### 2.2.2. Preparation of Magnetic Biocomposites

The F18 glass [21] was kindly provided by the VETRA–HighTech Bioceramics company, Ribeirão Preto-SP, Brazil. The glass frit was ground into a powder (average particle size ~5 μm) in the planetary ball mill (550 rpm/60 min). Then, the bioactive glass powder was mixed with the magnetic phase strontium-doped lanthanum manganite La_0.8_Sr_0.2_MnO_3_ at 5, 10, 20, and 30 wt% (denominated LSM5, LSM10, LSM20 and LSM30, respectively). These compositions were homogenized in the planetary ball mill with isopropyl alcohol at 150 rpm for 30 min. The powder mixture was uniaxially pressed into discs of ø 10 mm (~200 MPa), heated to 650 °C (heating rate of 10 °C/min) and cooled within the furnace.

### 2.3. Characterization

#### 2.3.1. Influence of LSM on the Sinterability of Bioactive Glass F18

The influence of LSM powder on the sinterability of F18 bioactive glass was studied by a heating stage microscope MISURA HSM ODHT 1400 (Expert System Solutions). To this end, cylindrical pellets (ø3 mm × 3 mm) were uniaxially pressed at 200 MPa. The green density of the pellets was approximately 55%. The sintering measurements were performed to a maximum temperature of 900 °C with a heating rate of 10 °C/min. The cross-section area image projected during the sintering was recorded every 1 °C and it was used to calculate shrinkage rate of the pellets. The density during the sintering process was calculated using Equation (1):

(1) $$\rho =\frac{h_{r}}{{A^{2}}_{r}}\;\rho_{0,}$$

where $\rho_{0}$ is the relative density of the green body, $h_{r}$ is the relative height of the sample calculated by the ratio with initial height (*h*/*h*_0_), and *A_r_* is the relative area of the sample calculated by the ratio with an initial area (*A/A*_0_) [22]. The theoretical density of the composites was calculated by the rule of mixtures, Equation (2), which considers the weight percentage and the density of the phases that form the composites. The weight percentage (*w*_i_) varies according to the composite composition and the densities (*ρ_i_*) of the phase F18 bioactive glass and the LSM are 2.6 g/cm^3^ and 6.5 g/cm^3^, respectively.

(2) $$\rho_{0}=\frac{1}{\sum_{i}\frac{wi}{\rho i}}$$

#### 2.3.2. Microstructure

The LSM pure magnetic powder and composite samples were 200-mesh-sieved to be used for various tests. The phase composition was analyzed by X-ray powder diffraction (XRD: Rigaku Ultima IV) with Cu K-alpha radiation (k = 0.15 nm, 40 kV, 20 mA). The HighScore Plus program was used for phase identification and the quantification was carried out by the Rietveld refinement method. A scanning electron microscope (SEM: Philips XL30 FEG, F.E.I. Company, Hillsboro, OR, USA) was used to observe the particle morphology. Particle size distributions (PSD) of F18 bioactive glass and LSM powders were measured by particle size analyzer (HORIBA LA-930). Anhydrous isopropyl alcohol (P.A.—99.5%) was used as liquid media, without any dispersant. 

#### 2.3.3. Magnetic Properties and Calorimetry under Alternating Magnetic Field

Magnetization measurements as a function of applied magnetic field (MxT) were performed using a vibrating sample magnetometer (VSM), by Quantum Design MPMS3 SQUID VSM, at three different temperatures (250, 300 and 350 K). The DC magnetic susceptibility measurements $\left(\chi_{DC}\left(T\right)=\frac{M}{H}\right)$ as a function of temperature, were performed using zero-field-cooling/field-cooling (ZFC/FC) for pure LSM phase and the LSM20 sample. In the ZFC/FC measurements, the sample was cooled from room temperature to 200 K, without a magnetic field (zero-field cooling), and then a magnetic field *H* = 100 Oe, 1 kOe, and 5 kOe was applied. $\chi_{DC}\left(T\right)$ was measured while the sample was heated with a rate of 2 K/min. Afterwards, the process was repeated, but during the cooling, a magnetic field was applied (field cooling) and $\chi_{DC}\left(T\right)$ was measured. This analysis allows one to accurately determine the Curie temperature (*T_c_*) and study the influence of temperature on the magnetic properties. 

**Figure 1:**
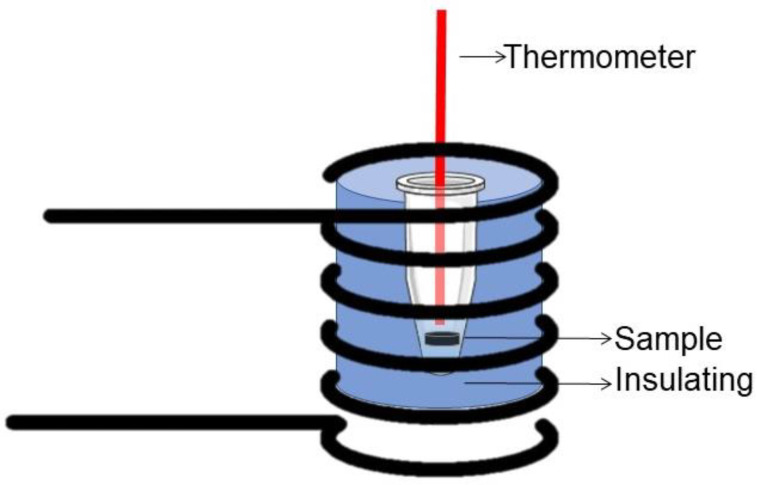
Schematic of the experimental setup for calorimetry measurements. The drawings were adapted from Ref. [23].

Calorimetry measurements were used to evaluate the composite heating ability when exposed to an alternating magnetic field (135 kHz, 100 Oe). Biocomposite bulk samples with dimensions of ø5 × 1 mm were inserted into plastic tubes (Eppendorf^TM^) with 200 mg of water and isolated with polystyrene foam. These samples were placed inside a solenoid coil which was 14 mm in diameter, 87 mm in length, had 15 turns and 1.1 µH of inductance (see Figure 1). Then, the initial temperature was stabilized at 24 °C and temperature rise was recorded using a fiber-optic thermometer (Qualitrol NOMAD-Touch Portable Fiber Optic Monitor, ± 0.5 °C). All measurements were carried out within a time period of 500 s. For more details about the hyperthermia system, readers are referred to reference [23].

After a long period of time, the temperature will tend to a constant maximum value called *T_max_.* This parameter can be obtained using Equation (3), which describes the variation of temperature as a function of time from the adjustment of the experimental data obtained [24]:

(3) $$T\left(t\right)=T_{0}+\Delta T_{max}\;\left(1-e^{-\frac{t}{\tau }}\right),$$

where $T_{0}$ is the initial temperature (24 °C) and ∆*T_max_* is the temperature change from the initial stage to a constant maximum value (steady state).

The specific absorption rate (*SAR*) is the amount of electromagnetic power absorbed per unit of mass, and it is described as the efficiency of the magnetic particles to produce heat in response to an external alternating magnetic field. The *SAR* value is affected by different factors, such as particle size, shape and magnetic properties, and magnetic field parameters, i.e., frequency and magnitude [15,25]. The *SAR* was calculated according to Equation (4), which was adapted from Iglesias [26], considering a non-adiabatic system and employing parameters associated with the energy flow between neighboring systems.

(4) $$T\left(t\right)=T_{0}+m_{p}\;\frac{SAR}{\alpha }\;\left(1-e^{\frac{-\alpha t}{C_{susp}}}\right),$$

where *C_susp_* is the heat capacity of aqueous dispersion (J/K), *m_p_* is the mass of magnetic particles (g), *T*_0_ is the initial temperature (°C), *t* is the exposure time, and α is the conductance, a parameter that measures the intensity of the interaction between neighboring systems. The heat capacity of water was taken as 4.2 J/g °C. To calculate the heat capacity of biocomposite samples, we used the rule of mixtures considering 0.66 J/g °C and 0.85 J/g °C, for LSM and the multicomponent F18 silicate glass, respectively. 

#### 2.3.4. In Vitro Bioactivity

The in vitro bioactivity test was obtained using a simulated body fluid (SBF) solution, according to the method proposed by Kokubo et al. [27]. Biocomposite bulk samples (~ø10 mm × 3 mm) were soaked in SBF at 36.7 °C under continuous shaking for various different times. These tests aimed to verify the rate of HCA layer formation in the sample surfaces. The samples were taken out from SBF after soaking from 24 h to 15 days, and then were carefully rinsed with acetone and dried at room temperature. After that, the samples were analyzed by using a scanning electron microscope (SEM, Philips XL30 FEG) and by FTIR (SPECTRUM GX-DE, Perkin-Elmer Co, Waltham, MA, USA), collected in the 4000–400 cm^−1^ range. Both techniques were used to identify different morphologies of the materials before and after soaking in SBF.

## 3. Results

### 3.1. Influence of LSM on the Sinterability of Bioactive Glass F18

**Figure 2:**
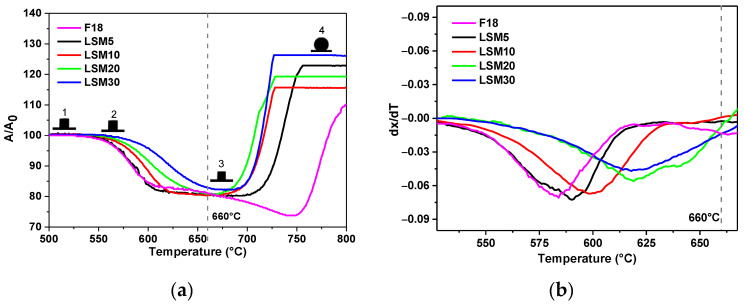
Sintering Curves of composites. (**a**) Sample area vs. Temperature (Shrinkage Curve) estimated errors F18 ± 0.6%/5LSM ± 2.7%/10LSM ± 1.1%/20LSM ± 4.0%/30LSM ± 0.5%. These images 1 to 4 represents the specific temperatures (1) Sintering Temperature, (2) Softening Temperature, (3) Maximum densification temperature and (4) expansion (**b**) Maximum densification rate vs. Temperature.

**Figure 3:**
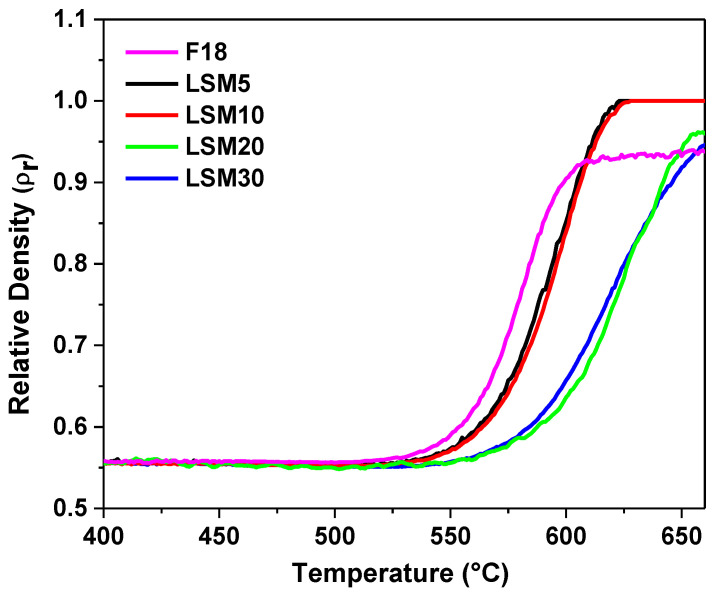
Relative density as a function of temperature of composites with a maximum temperature of 660 °C and heating rate of 10 °C/min.

Figure 2a shows the sintering curves of the composites and describes the main characteristic temperatures, such as sintering, softening, expansion, and melting. These temperatures are summarized in Table 1. The sintering temperature (660 °C) of the composites was determined by the sintering curve, corresponding to the maximum linear shrinkage for all samples. The F18 bioactive material is a crystallization-resistant glass; therefore, sintering by viscous flow will take place to completion before surface crystallization. This fact can be confirmed in Figure 3, where the relative density at saturation reached 1.0 for pure F18 bioactive glass. 

As the content of LSM is increased (Figure 2), the onset of sintering is slightly shifted to higher temperatures. The shrinkage rate also decreased, which was expected, as the LSM particles act as barriers for viscous flow. However, except for sample LSM30 (*A*/*A*_0_ = 18%), all the compositions reached a final shrinkage of ~20% at saturation. To ensure maximum densification of the composites during sintering, the derivative of the sintering curve (Figure 2b) was evaluated. The minimum point indicates the temperature at which the densification mechanism takes place with a maximum rate. The maximum densification temperature lies in the range of 575–625 °C. Thus, at the sintering temperature of 660 °C, all compositions pass through the maximum densification range. For temperatures above 700 °C, a significant expansion was observed for pure F18 glass and all other samples. This could be caused by the expansion of the entrapped air inside the sintered structure or perhaps degassing. Both of these mechanisms, isolated or combined, lead to the formation of bubbles and sample swelling. 

The calculated relative density curves are shown in Figure 3. All samples showed high density at saturation, with the presence of a single shrinkage stage. This behavior resulted from viscous flow sintering and is characterized by a sharp shrinkage in a short period. It can be observed that the compositions LSM5 and LSM10 showed maximum densification. The addition of LSM did not decrease the overall densification of the composite.

### 3.2. XRD and SEM

**Figure 4:**
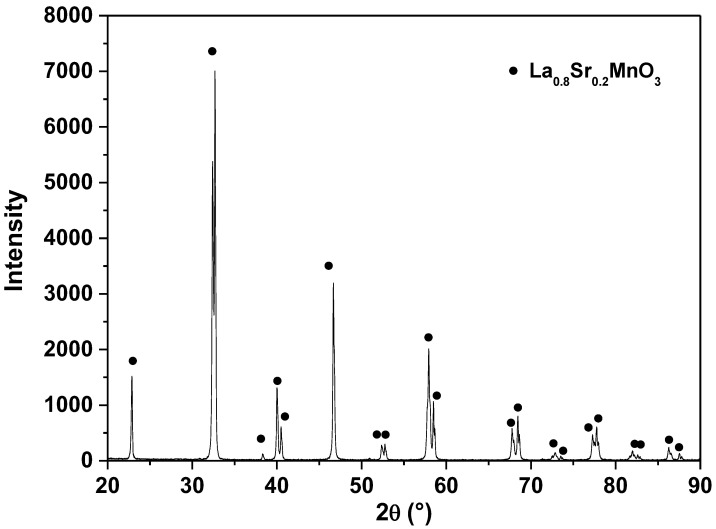
Powder X-ray diffraction pattern of the LSM particles.

Figure 4 shows the XRD pattern of the LSM particles. The magnetic phase was well crystallized, considering the position of diffraction peaks corresponding to the LSM standard diffractogram [ICSD 51655]; no signs of a secondary crystalline phase were detected. The XRD patterns were analyzed by Rietveld refinement to obtain the structural parameters. The calculated lattice parameters (a and c), unit cell volume (V), index R_wp_ and goodness of fit (S) are given in Table 2. The R_wp_ and S are Rietveld refinement parameters that indicate the relationship between calculated XRD intensities and experimental XRD intensities are considered satisfactory in a good refinement when the value is among 10% and 20%, and 1, respectively. The LSM particles present a single phase with no detectable impurities and they have a rhombohedral structure with the Rc3 (167) space group due to the replacement of La by Sr ions [28].

**Figure 5:**
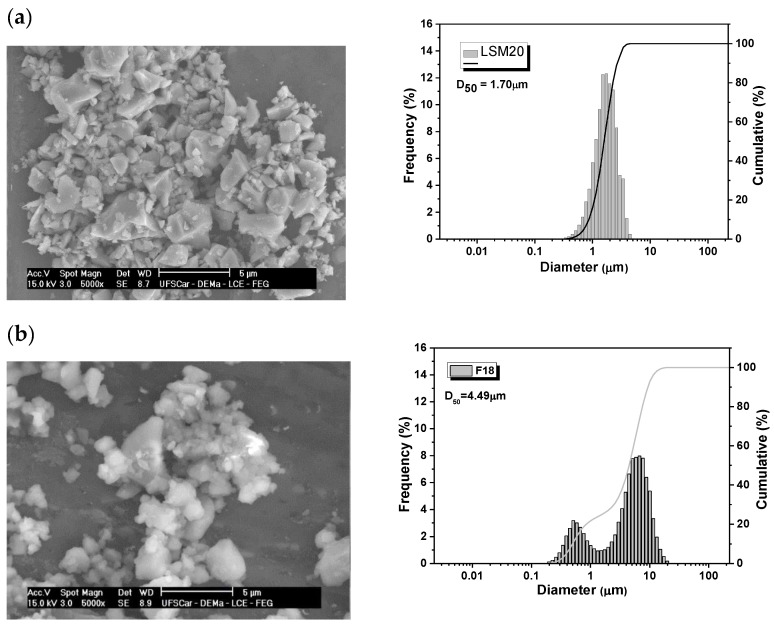
SEM images and the particle size distribution by diffraction laser (**a**) LSM particles (**b**) F18 particles.

The particle morphology and size distribution of the LSM and F18 bioactive glass are shown in Figure 5a,b, respectively. The particles have irregular shapes and large agglomerates that resulted from the grinding process (milling balls). The LSM particles have a monomodal particle size distribution, with an average particle size of 1.7 μm. On the other hand, F18 bioactive glass particles present an average particle size of 4.5 μm, with a bimodal particle size distribution. This difference is important for the encapsulating process of the LSM particles through the glass matrix during sintering by viscous flow.

**Figure 6:**
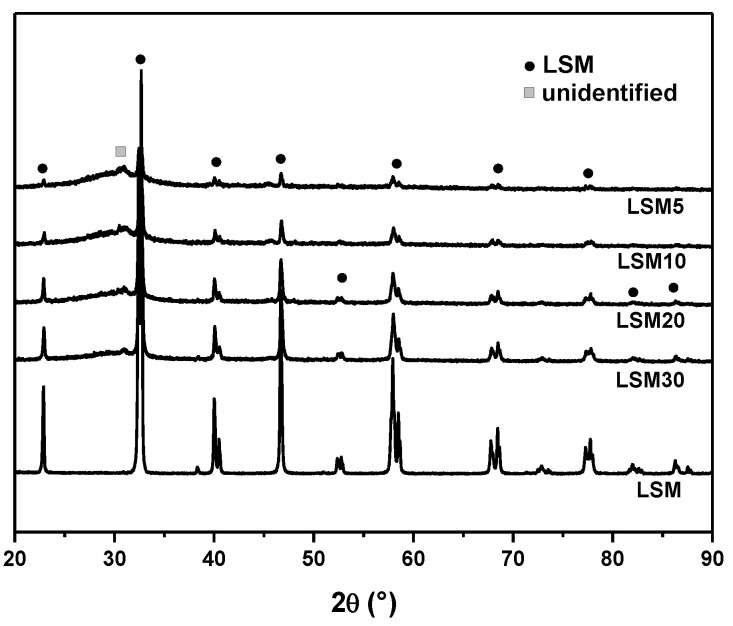
XRD patterns of the composites analyzed.

Figure 6 shows the XRD patterns of all composites to verify whether crystallization of the vitreous matrix occurred during the sintering process. The XRD patterns were similar, and only the magnetic crystalline phase LSM was identified in all samples. There were no signs of F18 bioactive glass crystallization. The bioactive glass F18 has greater stability and less tendency to crystallize compared to other bioglasses, allowing the sintering process to occur without forming surface crystals that would hinder the viscous flow process [30].

**Figure 7:**
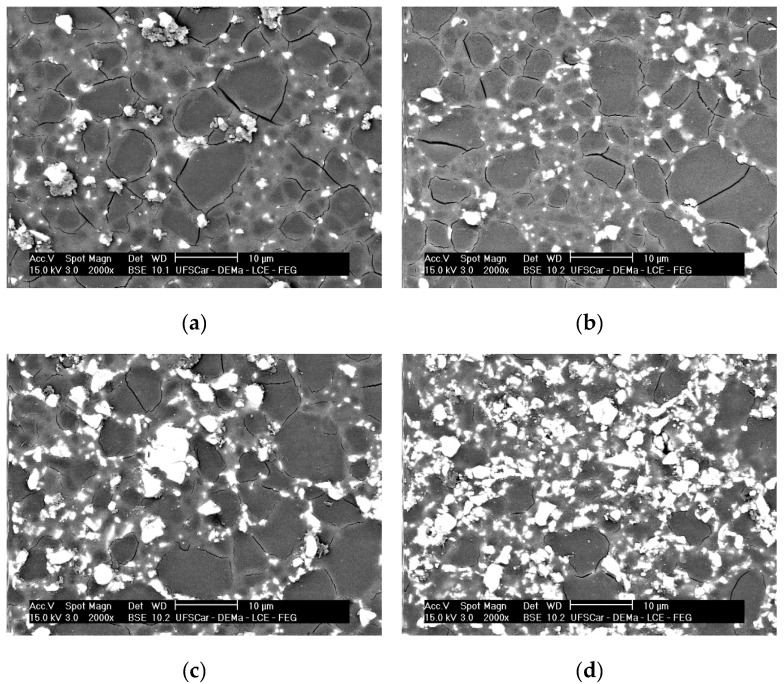
SEM images of polished surfaces of composites after chemical attack in a 70%HF/30%HCl solution. (**a**) LSM5; (**b**) LSM10; (**c**) LSM20; (**d**) LSM30.

Figure 7 shows the images obtained from the surface of all composites using the backscattered electron signal to increase contrast. The LSM particles are homogeneously dispersed in the glassy matrix and tend to concentrate on the grain boundaries, generally in the form of small agglomerates.

### 3.3. Magnetic Properties and Heating Efficiency under Alternating Magnetic Field

**Figure 8:**
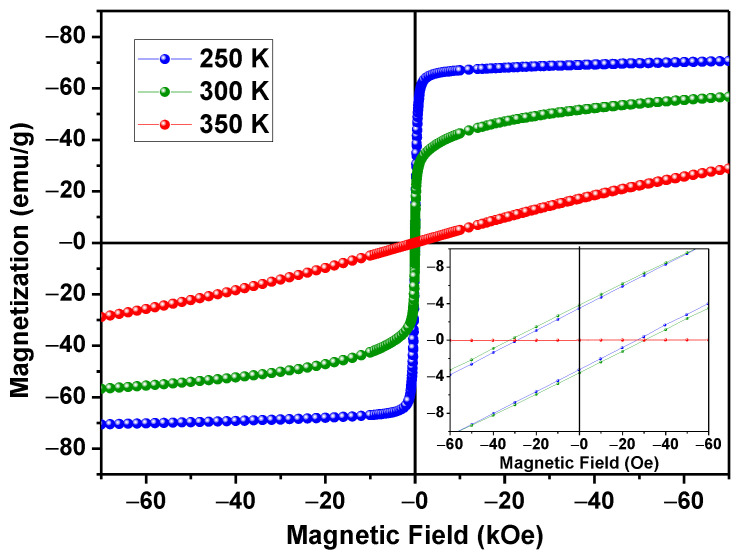
Magnetization as a function of the applied magnetic field of LSM particles at 250 K (blue), 300 K (green), 350 K (red)-accuracy 10^−8^ emu. The inset exhibits details of low fields.

The magnetization measurements, as a function of applied magnetic field of the sample (MxH) LSM at 250 K, 300 K, and 350 K, are shown in Figure 8. The saturation magnetization (M_s_), coercive field (H_c_), and remanent magnetization (M_r_) are summarized in Table 3. To compare the present results with other functional materials aimed at the same type of treatment, we have included the magnetic parameters of a previous paper [31]. The LSM exhibits a narrower magnetic hysteresis loop, which is representative of soft ferromagnetic materials with low coercivity and remanence at room temperature. Magnetic particles for biological applications are required to be soft magnets, which can be demagnetized with low coercive energy and retain some magnetization after removing the magnetic field. These properties make our material a suitable candidate for cancer treatment by magnetic hyperthermia, as this application requires a continuous magnetization process to generate heating. However, it is important to highlight that the magnetic properties of any ferromagnet depend on many factors, such as particle size, shape, crystalline defects, and surface effects. At 350 K, the LSM exhibits paramagnetic behavior without the presence of magnetic hysteresis, i.e., this corresponds to a temperature above the *Tc* of this material [32].

**Figure 9:**
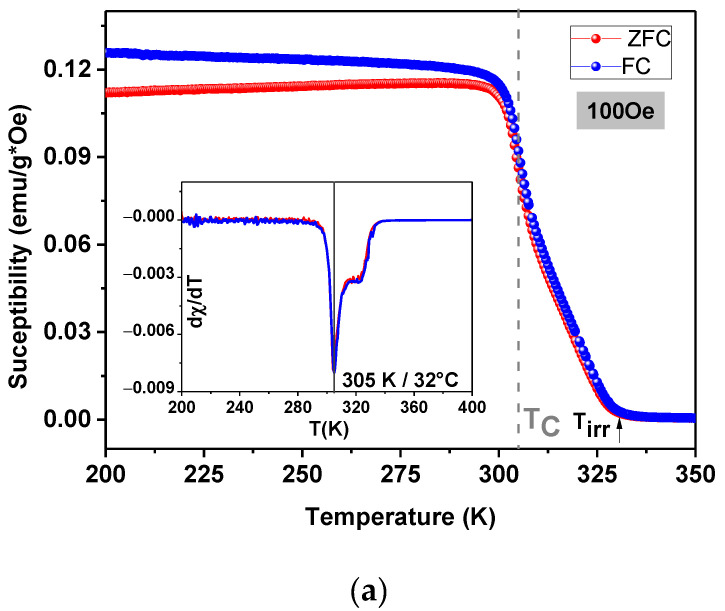

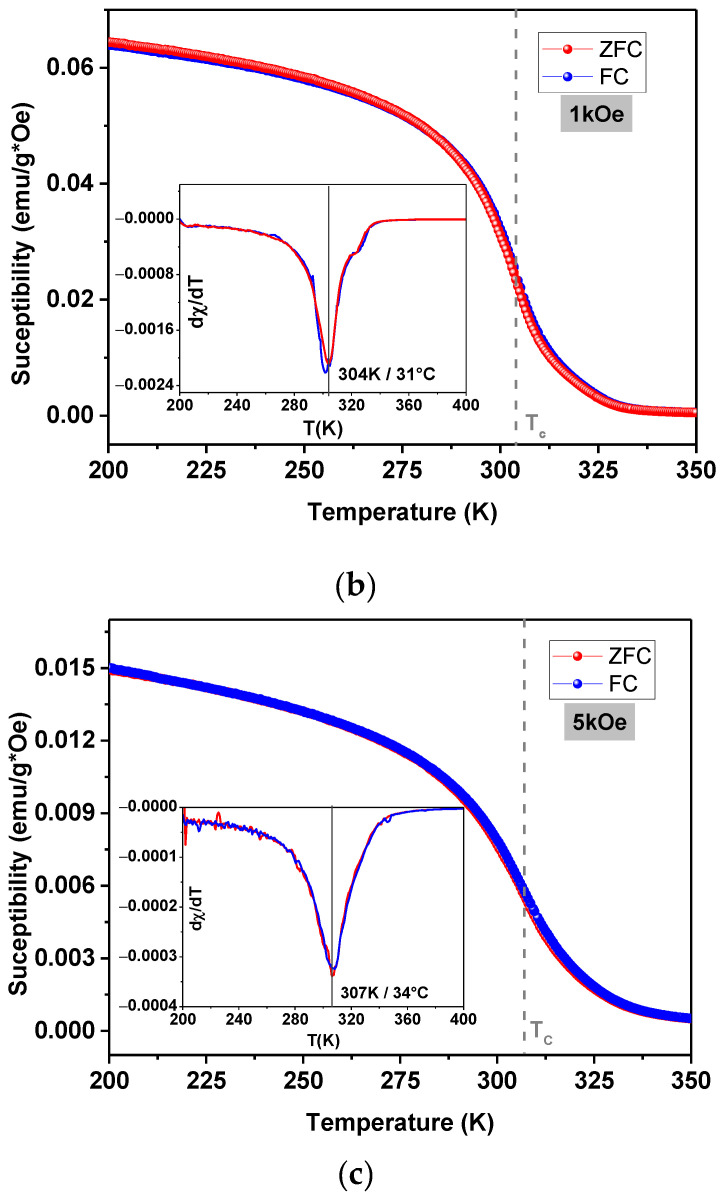
Temperature dependence of the magnetization (ZFC-FC) of the LSM sample for different magnetic fields accuracy 10^−8^ emu, and temperature uncertainty ± 0.5 K (**a**) 100 Oe; (**b**) 1 kOe; (**c**) 5 kOe.

Figure 9 presents magnetic susceptibility as a function of temperature $\left(\chi_{DC}\left(T\right)\right)$ using the ZFC/FC protocol LSM particles, for *H* = 100 Oe, 1 kOe and 5 kOe (Figure 9a–c, respectively). Figure 9 presents thermomagnetic irreversibility below *T_C_*_,_ between ZFC and FC, associated to competition between magnetocrystalline anisotropy and the magnetostatic energy that leads a separation of ZFC/FC curves. This behavior is expected due to the presence of multi-domain magnetic particles. At this temperature, the difference between the magnetocrystalline anisotropy and the magnetostatic energy is no longer null, so that to reduce the total energy, the formation of domain walls is favorable, forming multidomain structures [23,33].

The relationship between particle size and the magnetic properties of the ferromagnetic materials has been widely reported. The critical particle size of typical ferromagnets is <30 nm and this size indicates the transition from a single- to multidomain structure, which totally changes their magnetic behavior. The LSM average particle size is larger than 1 µm; considering a multidomain structure, its magnetization process is a result, mainly, of the movement of the domain walls [32,34,35].

The Curie temperature (*T_C_*) has been considered as the inflection point on the *χ* (*T*) curves, as shown in the inset in Figure 9. The LSM sample exhibits a ferromagnetic–paramagnetic transition (FM-PM) at 305 K (32 °C) and it is highlighted in the curves by a dashed line. This value is in agreement with the values reported in the literature [36]. However, it is worth mentioning that the variation in this value, depending on the magnitude of the applied field, results from the lack of structural homogeneity of the magnetic phases, which, in turn, affects the orientation of the moments with the application of the magnetic field [36,37].

There is a main peak and a “shoulder” in the *χ*(*T*) curves (see the inset in Figure 9a) that can be associated to the presence of more than one magnetic phase, which was not identified by XRD. The secondary phases may be the result of the formation of a magnetic phase with a more distinct stoichiometry than that of the La_0.8_Sr_0.2_MnO_3_ phase. Similar results were found in reference [38]. These measurements can be correlated with the particle size, size distribution, anisotropy energy, magnetic ordering or phase segregation. However, this fact is not clearly observed in Figure 9 b,c, due to the fact that for high fields, the magnetization of another phase was probably saturated. These figures also show small differences in *Tc*, calculated from the peaks of $\chi_{DC}\left(T\right)$ slopes. These differences are insignificant and associated with small variations in magnetization around *Tc*.

Above the Curie temperature, there is no magnetic hysteresis, but the paramagnetic phase exhibits a typical Curie–Weiss behavior $\left(\chi_{DC}\left(T\right)\propto \frac{1}{T}\right)$ due to the presence of non-ordered magnetic moments in the paramagnetic phase. Therefore, this movement of magnetic moments in the paramagnetic state must be considered because it can also generate magnetic losses in the form of heat above *Tc* [39].

**Figure 10:**
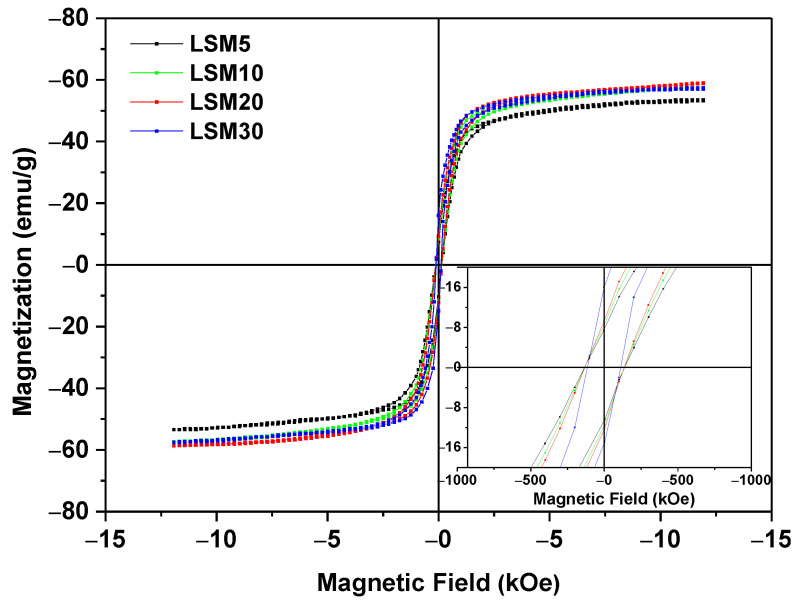
Magnetization of composites vs. magnetic field at 300 K normalized by mass of LSM-accuracy 10^−8^ emu.

**Figure 11:**
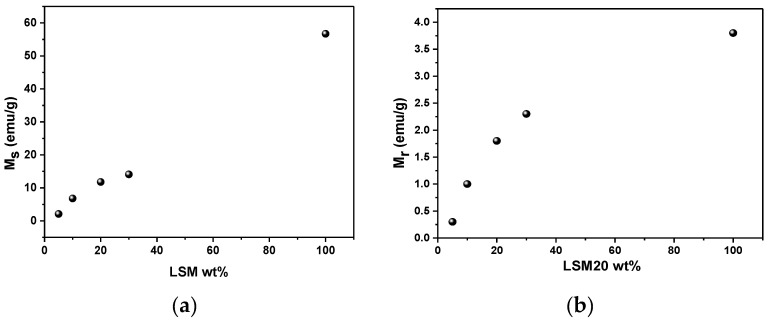
Saturation magnetization–M_s_ (**a**) and Remanence–M_r_ (**b**) as a function of the LSM amount in the composites, evaluated from M-H loop at 300 K-accuracy 10^−8^ emu. The value of 100% corresponds to the pure La_0.8_Sr_0.2_MnO_3_.

To understand the effect of the bioactive glass matrix on the magnetic properties of the LSM particles and evaluate the magnetic behavior of the composites, all MXH curves of composites at 300 K were normalized by the percentage of the LSM phase and are shown in Figure 10. As expected, the saturation magnetization (M_s_) and remanence magnetization (M_R_) do not change and present similar values (Figure 8), showing that the matrix does not contribute to the magnetic properties of the composite, as expected [40]. On the other hand, the coercivity of the samples does not change by increasing the LSM content in the composites. These composite magnetic parameters are shown in Figure 11a,b.

**Figure 12:**
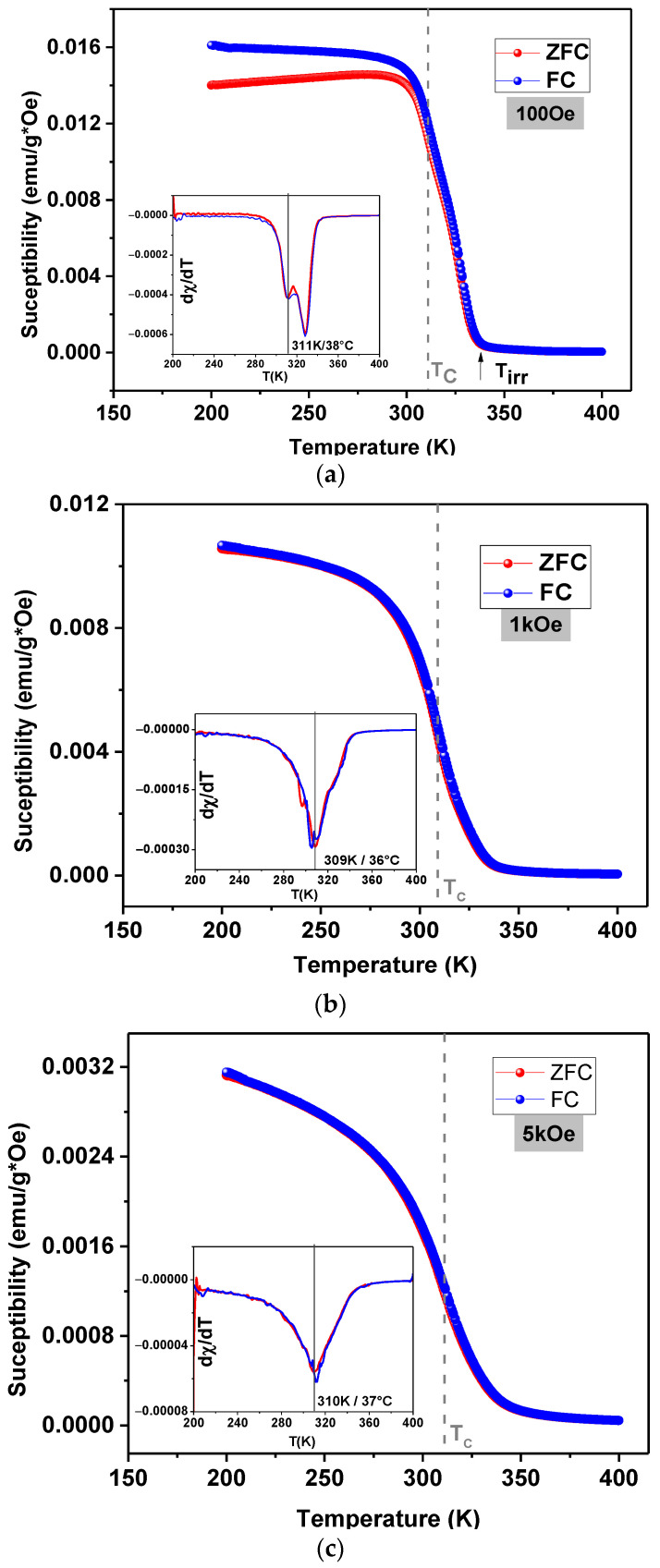
Magnetic susceptibility as a function of temperature using ZFC-FC protocol of the LSM20 sample for different magnetic fields accuracy 10^−8^ emu and ± 0.5 K (**a**) 100 Oe; (**b**) 1 kOe and (**c**) 5 kOe.

The field-cooled (FC) and zero-field-cooled (ZFC) *χ*(*T*) curves of the LSM20 composite are shown in Figure 12, for magnetic fields of 100 Oe, 1 kOe and 5 kOe. The *Tc* is highlighted on the ZFC-FC curves by a dashed line (Figure 12). This sample exhibits a Curie temperature at 311 K (38 °C), a value above that found for the pure LSM phase. Earlier reports suggest the variation in *Tc* is a result of residual stresses suffered by the magnetic particles generated during the sintering, due to the differences in the thermal expansion coefficient (TEC) of the main crystalline phase and the matrix. The CTE of the F18 matrix and the magnetic phase LSM are, respectively, 15.5 × 10^−6^ °C^−1^ and 11.4 × 10^−6^ °C^−1^ [41,42,43]. Hence, the LSM particles may be under residual stresses.

The magnetic behavior of the LSM phase is maintained in the LSM20 composite, even with the presence of the bioactive glass matrix. For low-magnitude fields (<100 Oe), the ZFC/FC curves separate at a critical temperature, called thermomagnetic irreversibility temperature (Figure 12a). In the same way as the pure phase LSM, above *Tc*, there is a small positive susceptibility. However, the susceptibility magnitude is lower considering the pure phase LSM due to the presence of the non-magnetic bioactive glass matrix.

**Figure 13:**
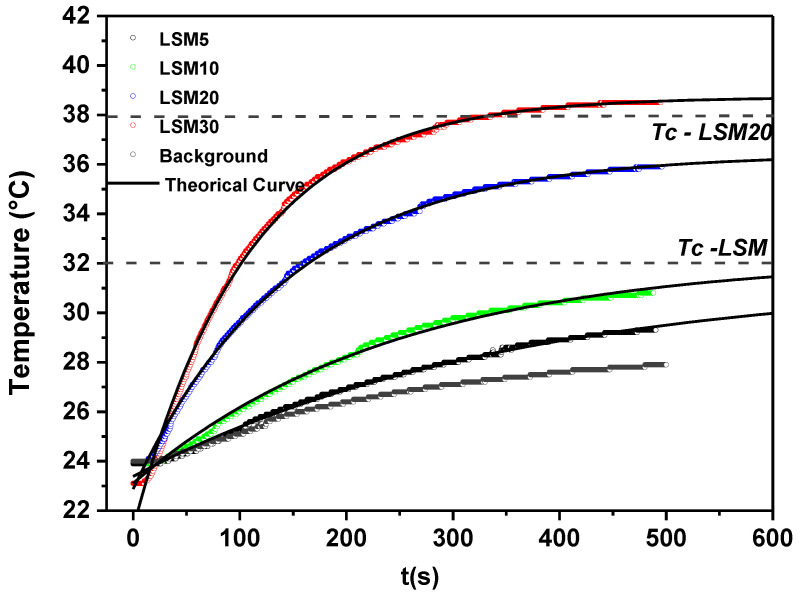
Temperature vs. heating time dependences for composite samples accuracy ± 0.5 °C.

Calorimetry measurements were carried out to evaluate the thermal response of the composites under the influence of an alternating magnetic field, with field strength and frequency of 100 Oe and 135 kHz, respectively (Figure 13). An aqueous suspension of composite particles was used as a sample. We observed that after activation of the external magnetic field, these composites show an abrupt temperature increase, which saturates after a certain time, depending on the LSM particle concentration in the composite. The saturation temperature is called *T_max_* and it is close to, but below, *Tc.*

Hysteresis loss is the main loss process attributed to ferromagnetic particles above its critical diameter in magnetic materials with a multidomain structure. The heating is related to the area of hysteresis over a complete magnetization cycle, which happens due to various factors, such as defects in the crystal structure, movements of domain walls, anisotropy and frequency of the alternating magnetic field. In general, these mechanisms transform the magnetic energy to thermal energy under the influence of an alternating magnetic field and its efficiency is measured in terms of the specific absorption rate (*SAR*) [24].

**Figure 14:**
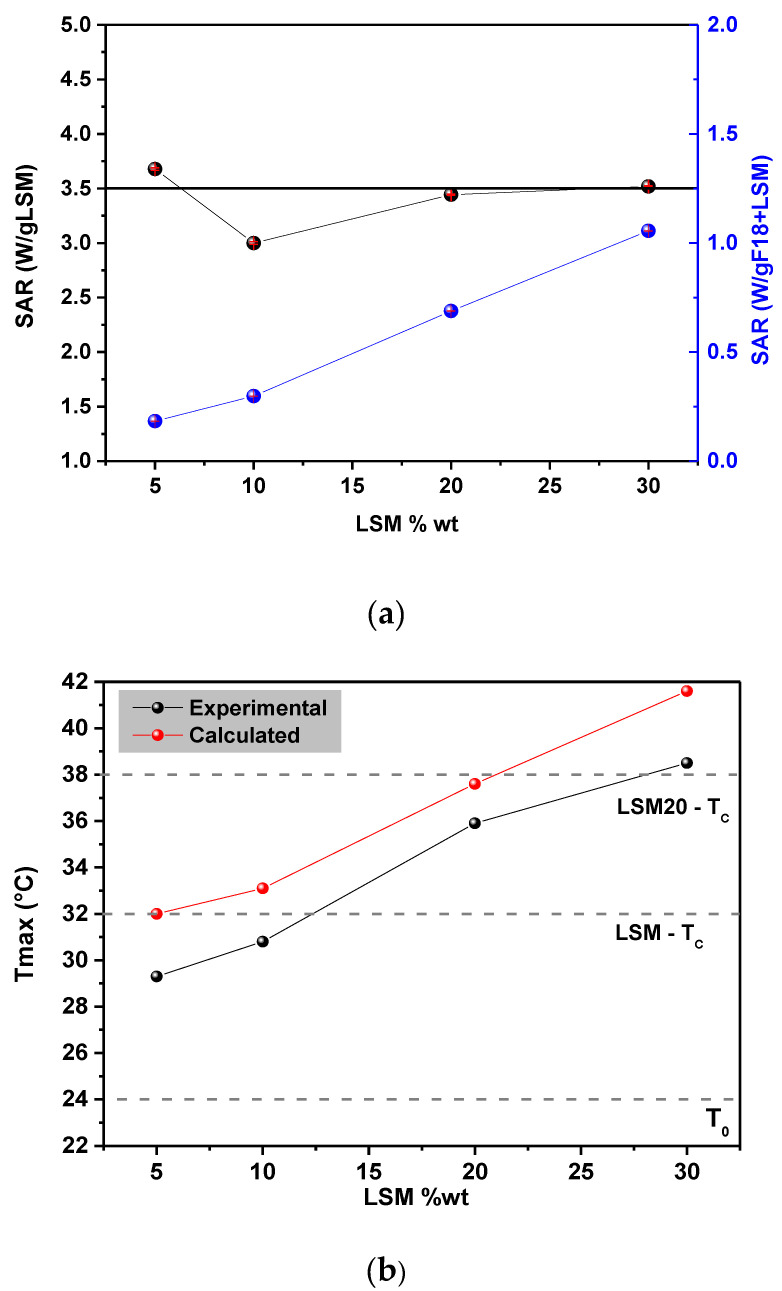
(**a**) *SAR* value obtained from experimental data using Equation (4) normalized by the mass of LSM in the composite. The inset is *SAR* value considering the mass of the composite components F18 + LSM. (**b**) Comparison between *T_max_* obtained by experimental data and *T_max_* calculated by Equation (3).

The *SAR* value was estimated from the heating data using Equation (4) (Figure 14a). The *SAR* value was normalized by the mass of LSM and it has an estimated value of 3.5 W/g LSM. The fluctuations among the values obtained for the different compositions could be attributed to inaccuracies in the mass concentrations of LSM during the manufacture of composites. Considering the total mass of the composite, i.e., including the mass of F18 glass, the *SAR* value increased with the content of LSM (see in Figure 14a). The temperature increases with the addition of the LSM phase concentration, from initial temperature until T_max_. Even with the similar *SAR* value, *T_max_* achieved during the calorimetry test is different for composites because this behavior is related to the area of interaction between LSM-matrix constituent systems. For a higher LSM content, two effects are expected: (1) the particles interact with each other with the application of the magnetic field and (2) the heat transfer becomes more effective due to the larger contact area with the matrix.

Figure 14b compares the *T_max_* values obtained experimentally and calculated by Equation (3). The experimental *T_max_* is close to *Tc*, and it presents values below the calculated *T_max_*. This happens because *T_max_* depends on both the features of heat exchange with environment and magnetic parameters of particles; in particular, on the disperse in the values of magnetization and Curie temperature [11]. Both calculated and experimental maximum temperature increase (Δ*T*) for each composition is shown in Table 4. As can be seen, Δ*T* is of approximately 5 °C for the composite containing 5 wt% of LSM and of 15 °C for the composite containing 30 wt% of LSM.

The maximum temperature achieved was 39 °C, for the LSM30; however, to be effective in cancer hyperthermia, the active material must provide a local temperature rise up to 42–43 °C. In the case of the LSM-F18 glass composites, there are two plausible alternatives: to increase the LSM content or alter the LSM stoichiometry to obtain a magnetic phase with slightly higher *Tc* due to a decrease in the magnetization of the particles with increasing temperatures near *Tc*, which results in a decrease in heat generation.

When developing a new material intended for cancer hyperthermia, not only the *Tc* of the magnetic phase, but also the following aspects must be considered: (1) the amount of the magnetic phase present within the composite; (2) the average particle size of the magnetic phase; (3) the thermal conductivity of the matrix; (4) the wettability of the magnetic phase by the glass during sintering; (5) the difference in thermal expansion coefficients between the magnetic phase and the matrix, whether resulting or not in residual stresses. All the aforementioned aspects may affect the overall *Tc* of the composite and, therefore, *SAR* and *T_max_*.

Another aspect to be considered is the magnetic field parameters that could be used in real therapy. Our findings are in agreement with the recently published results by Shlapa et al. [11]. Both *SAR* and *T_max_* are very sensitive to the magnetic field parameters used.

**Figure 15:**
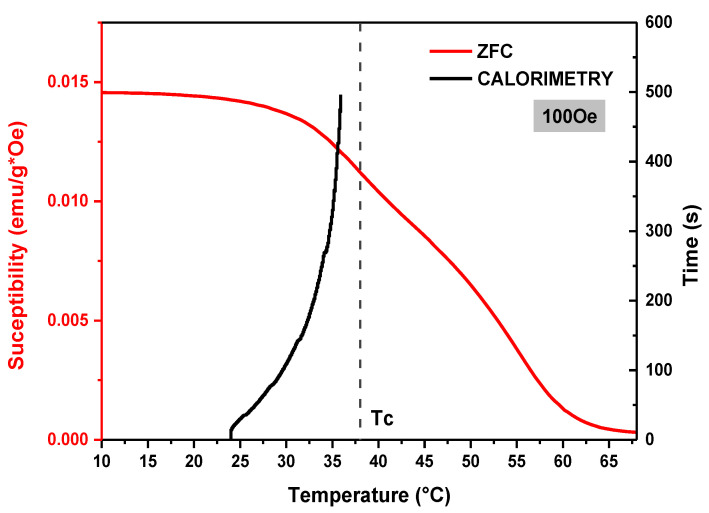
Thermal behavior of the LSM20 composite around *Tc* with a magnetic field = 100 Oe.

Figure 15 shows the relationship between the magnetic susceptibility and the magnetocaloric behavior, as a function of temperature for the LSM20 composite. Magnetic susceptibility is a measure of how much material may be magnetized when submitted to an applied magnetic field, and it is described by Curie’s Law $\left(\chi_{DC}\left(T\right)=\frac{M}{H}\right)$. When the material is heated, the magnetization becomes inversely proportional to the temperature and then decreases drastically with the temperature rise from the heating capability of the particles.

For MHT applications, using the magnetic material inside the body with a temperature ~36 °C, is a very sensitive phenomenon, mainly due to the smaller magnetization of the particles next to the Curie temperature. Hence, it should be considered for the design of new materials for self-controlled magnetic hyperthermia.

### 3.4. In Vitro Bioactivity

**Figure 16:**
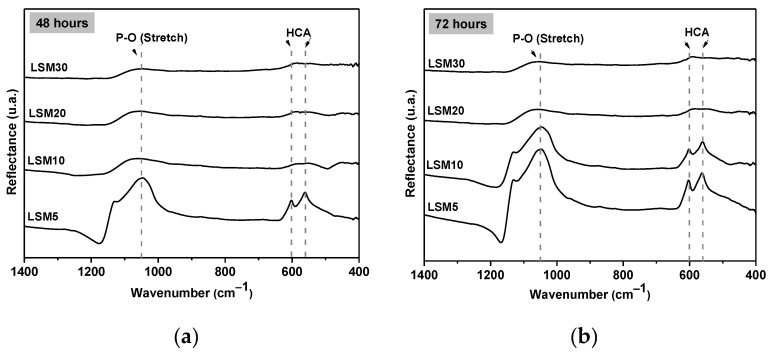

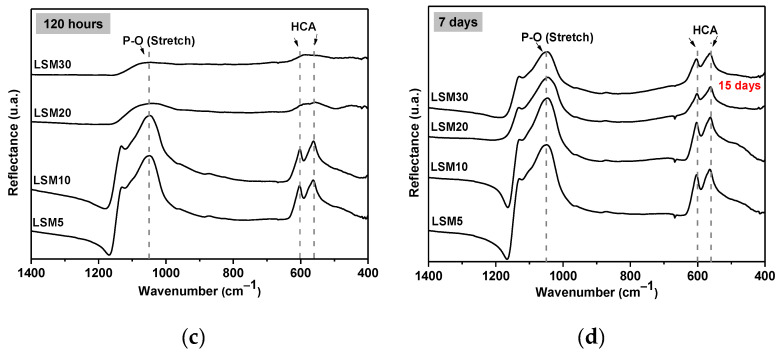
Infrared spectroscopy (FTIR) for the composite samples after SBF soaking (HCA peaks highlights by black arrows). (**a**) 48 hours, (**b**) 72 hours, (**c**) 120 hours, (**d**) 7 days with exception of LSM30 by 15 days.

An ideal material for treating bone cancer by hyperthermia would comprise a double function: to kill the cancer cells trough heating in the first stage of the treatment and to promote healthy bone cell growth in a second stage. Marin et al. [44] demonstrated the capacity of the F18 bioactive glass in stimulating osteogenesis; in other words, it has the ability of regenerating bone tissue, which is very important for composite application. The in vitro bioactivity or the apatite formation ability of the composites was evaluated by an in vitro bioactivity test, using Kokubo’s SBF-K9 solution. As shown in Figure 16, infrared spectroscopy analysis detected hydroxycarbonate apatite (HCA) layer formation after 48 h for the LSM5 samples (Figure 16a) and after 15 days for the LSM30 (Figure 16d). The presence of an HCA layer was confirmed by the presence of three main peaks at approximately 1050 cm^−1^ (P-O stretch) and at 602 and 590 cm^−1^ (P-O bend) [21,45]. The three peaks are indicated by black arrows in Figure 16.

In general, by increasing the concentration of LSM, the onset of HCA crystallization shifts to longer times than pure F18 (onset~12 h [15]). This behavior was already expected, since, with the increase in the concentration of the magnetic phase, there is a reduction in the area of the glass on the surface of the composite exposed to the SBF solution, reducing the leaching of the ions necessary for the formation of the HCA layer.

**Figure 17:**
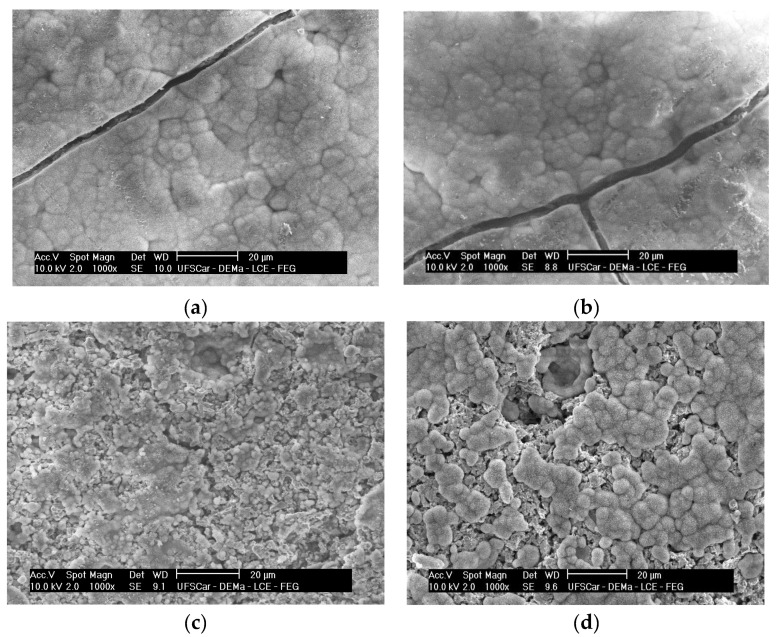
SEM images of the composites after SBF soaking by 7 days (**a**) LSM5, (**b**) LSM10, (**c**) LSM20 and 15 days for (**d**) LSM30.

Figure 17 shows the SEM images of all sintered samples after soaking in SBF for 7 days. There is an HCA layer covering the surface of all samples after exposure to SBF. These globular formations, comprising intertwined HCA acicular crystals, are a crystalline habit commonly observed after the in vitro test. According to Souza et al. [21], with the increase in the bioactive glass exposure time to the SBF-K9 solution, there is an increase in the size of the globular HCA structures over its surface. This can be noted in the cases of the LSM5 and LSM10 composites, where globular structures were increased. The only exception was the LSM30 sample, where the surface was not completely covered by an HCA layer, even after 15 days (Figure 17d).

In terms of apatite formation ability, the performance of these composites is better than other bioactive glasses and glass ceramics, except for the gold standard 45S5 bioglass and biosilicate glass ceramic found in the literature [46].

## 4. Conclusions

In this study, bioactive magnetic composites were developed through the sintering of F18 bioactive glass, containing gradual additions of a strontium-doped lanthanum manganite (La_0.8_Sr_0.2_MnO_3_–LSM). ZFC/FC curves showed that the pure LSM phase has a *Tc* of ~32 °C. However, when the LSM particles are constrained in the F18 bioglass matrix, the *Tc* is increased to ~37 °C (composition LSM20). Both saturation magnetization and remanence increased with the LSM20 content, although not linearly. Calorimetry evaluation in aqueous medium revealed that the composites exhibit a fast temperature increase with time, reaching saturation within 5–8 min, depending on the LSM20 content. The measured temperature increases under an external magnetic field (Δ*T*) ranged from 5 °C (LSM5) to 15 °C (LSM30). The magnetic susceptibility decreased drastically with increasing temperature, which, in turn, saturated at 2–3 °C below the *Tc*. The calculated values for the specific absorption rate were smaller than that estimated for the pure LSM phase (3.5 W/g), lying between 1.4 W/g (LSM5) and 3.0 W/g (LSM30).

As proven by in vitro tests, all the composites in this work showed significant apatite formation ability; however, the addition of LSM increased the onset time for HCA formation, from 2 days (composition LSM5) to 15 days (LSM30). The best composite reached 40 °C in 500 s, which is quite close to the desired tumor treatment temperature (42 °C). This result demonstrates the proof of concept sought in this research. Therefore, these materials show great potential to be used as smart bioactive bone grafts in patients affected by bone tumors and warrant further development.

## Figures and Tables

**Table 1:** Sintering Temperature (*T_s_*), Softening Temperature (*T_a_*), Maximum densification temperature (*T_max_*) in sintering saturation. The typical error in these temperatures is ±5 °C.

Composition	*T_s_* (°C)	*T_a_* (°C)	*T_max_* (°C)
F18	571	571	650
LSM5	574	574	650
LSM10	582	582	655
LSM20	602	616	658
LSM30	603	605	660

**Table 2:** Crystallographic parameters of La_0.8_Sr_0.2_MnO_3_ particles.

Parameters	Rietveld	ICSD 51655 *
D(g/cm^3^)	6.52	6.56
a (nm)	0.5526	0.5518
c (nm)	1.3362	1.3344
V (nm^−3^)	0.35335	0.35180
R_wp_ (%)	11.35	-
S	1.68	-

* Data from [29].

**Table 3:** Magnetic parameters of LSM particles (accuracy 10^−8^ emu).

Material	*T* (K)	M_s_ (emu/g)	M_r_ (emu/g)	H_c_ (Oe)	M_r_/M_s_
LSM, average particle size~1.7 μm	250	70.6	3.4	~30	0.05
	300	56.7	3.8	~30	0.08
Magnetite, average particle size~5 μm. Data from [31]	300	58.8	3.8	~150	0.23

**Table 4:** Calculated and experimental temperature increase for the tested composites.

LSM Content (wt%)	Calculated Temperature Increase–Δ*T* (°C)	Experimental Temperature Increase–Δ*T* (°C)
5	8	5
10	9	7
20	14	11
30	18	15

## Data Availability

Not applicable.
